# 

**Published:** 1868-02-01

**Authors:** 


Vol. XXV. Number 3.

### THE CHICAGO MEDICAL JOURNAL.

*PUBLISHED SEMI-MONTHLY.*

EDITED BY

J. Adams Allen, M.D., LL.D.

FEBRUARY 1, 1868.

CHICAGO:

Church, Goodman and Donnelley, Steam Book and Job Printers,

108 and 110 Dearborn Street.

